# RAMRSGL: A Robust Adaptive Multinomial Regression Model for Multicancer Classification

**DOI:** 10.1155/2021/5584684

**Published:** 2021-05-25

**Authors:** Lei Wang, Juntao Li, Juanfang Liu, Mingming Chang

**Affiliations:** ^1^Department of Basic Science Teaching, Henan Polytechnic Institute, Nanyang, 473000 Henan, China; ^2^College of Mathematics and Information Science, Henan Normal University, Xinxiang, 453007 Henan, China

## Abstract

In view of the challenges of the group Lasso penalty methods for multicancer microarray data analysis, e.g., dividing genes into groups in advance and biological interpretability, we propose a robust adaptive multinomial regression with sparse group Lasso penalty (RAMRSGL) model. By adopting the overlapping clustering strategy, affinity propagation clustering is employed to obtain each cancer gene subtype, which explores the group structure of each cancer subtype and merges the groups of all subtypes. In addition, the data-driven weights based on noise are added to the sparse group Lasso penalty, combining with the multinomial log-likelihood function to perform multiclassification and adaptive group gene selection simultaneously. The experimental results on acute leukemia data verify the effectiveness of the proposed method.

## 1. Introduction

With the development of technology, the scale of data is constantly increasing, and the dimension of data is rapidly expanding. Data as diverse as transaction data, user rating data, Web usage data, gene expression data, and multimedia data can have hundreds or thousands of dimensions and even more [1]. Therefore, the microarray data has the characteristics of small sample and ultrahigh dimension [2]. The birth of microarray data makes it possible to diagnose complex diseases such as cancer at the genetic level. In the process of using gene expression data for diagnosis, genes are treated as characteristics or attributes, and tissue samples are labeled as specific types, such as tumor tissue or normal tissue, various subtypes of cancer. The classifier is then constructed using machine learning methods to predict the types of the new sample [3–6]. However, only a few genes are closely related to cancer diagnostic tasks for the microarray gene expression data. Therefore, although the classification process is completely consistent with the traditional data, cancer classification based on the gene expression data still faces great challenges [7, 8].

Due to the characteristics of automatic variable selection, sparse regression methods [9–13] have attracted a surge of attention in cancer diagnosis and gene selection. To tackle the problem that the *l*_1_ regularization has a biased gene selection and does not have the oracle property, Wu et al. [13] in 2018 have investigated *l*_1_/*l*_2_ regularized logistic regression for gene selection in high-dimensional cancer classification. In terms of classification performance, the experimental results on three DNA microarray datasets demonstrate that the proposed method outperforms other commonly used sparse methods. To address the problem that there are high correlations among genes, a two-stage sparse logistic regression has been proposed by Algamal and Lee [12] in 2019, which is aimed at obtaining an efficient subset of genes with high classification capabilities by combining the screening approach as a filter method and adaptive Lasso with a new weight as an embedded method. The experimental results demonstrate that the top selected genes are biologically related to the cancer type, which is useful for cancer classification using DNA gene expression data in real clinical practice. To handle the group structures of the time-dependent clinical variables in the model, Zhang et al. [10] in 2020 have developed a high-dimensional logistic regression and introduced the group spline-penalty or group smooth-penalty. This method is easy to implement since it can be turned into a group minimax concave penalty problem after certain transformations.

Yuan and Lin [14] first proposed a group Lasso regression model using *l*_2_-norm penalty. Group Lasso [15–17] can generate interpopulation sparsity, i.e., automatic identification of important gene groups. To identify several important genes but not all genes in the same group, Simon et al. [18] proposed the sparse group Lasso. Since both the *l*_1_-norm penalty and the *l*_2_-norm penalty are introduced into the model, it can generate both intergroup sparsity and intragroup sparsity. By introducing the weighted gene coexpression network analysis and information theory into the sparse group Lasso, Li et al. [19] proposed three criteria for evaluating the importance of genes within the population and then proposed the adaptive sparse group Lasso model.

Generally, the group Lasso methods rely on early grouping, so it is important to choose an appropriate grouping method. To this end, various algorithms have been proposed. Clustering, the most popular, has been used since the first gene expression dataset is born and is still the most widely used [20, 21]. Furthermore, clustering methods can be divided into four categories: prototype-based clustering [22, 23], density-based clustering [24, 25], hierarchical clustering [26, 27], and spectral clustering [28]. For gene expression data, Sharan et al. [29] proposed a clustering algorithm by linking kernels, which is named CLICK. The CLICK algorithm uses graph theory and statistical techniques to identify tight groups of highly similar elements and then uses some heuristic process to extend kernel extensions into modules. Weighted gene coexpression network analysis (WGCNA) [30] is a clustering method developed for microarray data, which improves the classical bottom-up clustering algorithm. Instead of slicing a tree at a certain height like traditional hierarchical clustering, WGCNA uses a dynamic tree slicing algorithm to ensure that the resulting clustering meets several criteria related to cohesion and separation. Affinity propagation (AP) is a spectral-based clustering algorithm proposed by Frey and Dueck in 2007 [31]. AP clustering takes the similarity measure between data points as input and then automatically identifies a group of high-quality clustering centers and corresponding clusters through the continuous transmission of two kinds of real value information between data points.

It is necessary to construct a robust sparse regression model for high-dimensional data with noise [32–35]. Traditional least square methods may not produce reliable estimators, while the least absolute deviation (LAD) estimator is an effective robust regression method. Wang et al. [36] proposed LAD-Lasso by combining LAD and Lasso, which can not only estimate parameters and select variables at the same time but also has strong resistance to heavy tail error or response outliers. In addition, the parameters of LAD-Lasso are easy to estimate and have oracle properties. However, LAD loss is not applicable for small residuals, especially when there is no heavy tail error and no outliers, the estimator shows poor performance. To improve on that, Lambert-Lacroix and Zwald [37] proposed a robust regression model combining Huber criterion and adaptive Lasso penalty. Huber criterion is also an effective method of robust regression. It is a mixture of the square error of relatively small error and the absolute error of relatively large error, which makes the model have good performance regardless of the size of residuals. To alleviate the influence of conversion parameters on the performance of Huber Lasso, Zheng et al. [38] proposed a convex combination of adaptive Lasso and LAD-Lasso with data-driven power, namely, robust adaptive Lasso (RA-Lasso).

To solve multiclassification problems effectively, Vincent and Hansen [39] introduced sparse group Lasso penalty into multinomial log-likelihood function, proposed multinomial sparse group Lasso model, and developed the solution algorithm. Due to this model adopts the method of random grouping, the obtained groups are not of biological significance. Li et al. [19] used WGCNA to cluster genes in advance, introduced a clustering method that could better explain the structure of genes, and then proposed an adaptive multinomial regression model with sparse overlap group Lasso penalty. Although these methods [40, 41] can solve the multiclassification problems in cancer diagnosis well, how to build a robust multinomial regression model for noisy data and how to use the noise information to construct data-driven weights so as to further increase the robustness of the model are problems that need to be solved.

In this paper, to obtain biologically significant gene clusters for each cancer subtype, AP clustering is used to cluster the three acute leukemia subtypes in advance on the noise-removed data. Then, the noise matrix is used to construct data-driven weights, based on which an adaptive sparse group Lasso penalty for multicancer microarray data is proposed. Furthermore, a robust adaptive multinomial regression model with sparse group Lasso penalty (RAMRSGL) is proposed based on log-likelihood loss, and a regularization solution algorithm is developed.

The structure of the rest paper is as follows: in Section 2, we first define the multiclassification problem and then elaborate the RAMRSGL model.  Section 3 verifies the effectiveness of the proposed model through experiments.  Section 4 eventually summarizes the whole paper.

## 2. Problem and Method

### 2.1. Preliminaries

Since a cancer often has different subtypes, cancer diagnosis requires not only determining whether a patient has cancer but also accurately identifying the type of cancer they have. As a result, cancer diagnosis can be modeled as a multiclassification problem. Suppose cancer has *K*(*K* ≥ 3) subtypes and a gene expression dataset *𝒟* = {(**x**_1_, *y*_1_), (**x**_2_, *y*_2_), ⋯, (**x**_*N*_, *y*_*N*_)} contains *N* samples, where **x**_*i*_ ∈ ℝ^*M*^ and *y*_*i*_ ∈ {1, 2, ⋯, *K*} are the gene expression sample and its label, respectively. For notation convenience, let **X** = (**x**_1_, **x**_2_,⋯,**x**_*N*_)^*T*^ and **y** = (*y*_1_, *y*_2_,⋯,*y*_*N*_)^*T*^ denote the sample matrix and its corresponding label vector, respectively. To identify the type of new sample **x**, we need to construct a decision function *f*(**x**) with *K* discriminant functions *f*_*k*_, i.e.,

(1) $$\begin{array}{c} f\left(x\right)=\underset{k}{\arg\;\max}f_{k}\left(x\right). \end{array}$$

Generally, linear discriminant function *f*_*k*_(**x**) = *β*^(*k*)^^*T*^**x** + *β*_0_^(*k*)^ is most widely used. Therefore, the construction of the decision function is always transformed into the problem of solving the optimal parameters *β*^(*k*)^ and *β*_0_^(*k*)^ of each discriminant function.

The above regression coefficients can be usually solved by the following Lasso model [42]:

(2) $$\begin{array}{c} \underset{\beta }{\min}\frac{1}{2}{\left\|y-X\beta \right\|}_{2}^{2}+\lambda {\left\|\beta \right\|}_{1}, \end{array}$$

where *λ* ≥ 0 is the regularization parameter. By using the *l*_1_-norm penalty, some coefficients that correspond to the features can be reduced to zero. To select features in groups, Yuan and Lin [14] proposed group Lasso (GL) in 2006.

(3) $$\begin{array}{c} \underset{\beta }{\min}\frac{1}{2n}{\left\|y-\overset{c}{\underset{l=1}{\sum }}\;X^{\left(l\right)}\beta^{\left(l\right)}\right\|}_{2}^{2}+\lambda \overset{c}{\underset{l=1}{\sum }}\;\sqrt{m_{l}}{\left\|\beta^{\left(l\right)}\right\|}_{2}, \end{array}$$

where **X**^(*l*)^ and *β*^(*l*)^ are the subset of the *l*-th group, and *m*_*l*_ represents the number of the *l*-th group. To generate both intergroup sparsity and intragroup sparsity, Simon et al. proposed sparse group Lasso (SGL) [18] in 2013.

(4) $$\begin{array}{c} \underset{\beta }{\min}\frac{1}{2n}{\left\|y-\overset{c}{\underset{l=1}{\sum }}\;X^{\left(l\right)}\beta^{\left(l\right)}\right\|}_{2}^{2}+\left(1-\alpha \right)\lambda \overset{c}{\underset{l=1}{\sum }}\;\sqrt{m_{l}}{\left\|\beta^{\left(l\right)}\right\|}_{2}+\alpha \lambda {\left\|\beta \right\|}_{1}, \end{array}$$

where 0 ≤ *α* ≤ 1 is also the regularization parameter. SGL penalty is a convex combination of Lasso penalty and group Lasso penalty, which can achieve two kinds of sparsity simultaneously. To achieve adaptive population gene selection, Li et al. proposed adaptive sparse group Lasso (ASGL-CMI) in 2017.

(5) $$\begin{array}{c} \underset{\beta }{\min}\frac{1}{2n}{\left\|y-\overset{c}{\underset{l=1}{\sum }}\;X^{\left(l\right)}\beta^{\left(l\right)}\right\|}_{2}^{2}+\left(1-\alpha \right)\lambda \overset{c}{\underset{l=1}{\sum }}\;\sqrt{m_{l}}{\left\|\beta^{\left(l\right)}\right\|}_{2}+\alpha \lambda {\left\|W\beta \right\|}_{1}, \end{array}$$

where **W** is the weight constructed based on information theory. The ASGL-CMI can adaptively select the important genes in the selected population by introducing the weight with biological significance.

The real gene expression datasets often have some missing values and contain noise, while the current models mostly ignore the point. Therefore, this paper is devoted to establish a robust classification model for gene expression data with noise and effectively identify the important genes related to cancer.

### 2.2. Robust Adaptive Multinomial Regression with Sparse Group Lasso Penalty

First, the input sample matrix is decomposed through robust principal component analysis. Then, the overlapping clustering strategy is adopted to cluster the genes on the leukemia data with noise removed by AP clustering, and the weight is constructed by using the noise matrix. Finally, the RAMRSGL model is constructed according to the clustering results and weight.

#### 2.2.1. Robust Principal Component Analysis

It is assumed that the gene expression data **X** conforms to the noise distribution, and the noise is usually sparse. As a modification of the widely used statistical procedure of principal component analysis (PCA), the robust principal component analysis (RPCA) works well with respect to grossly corrupted data [43]. Therefore, **X** can be decomposed into a low-rank matrix **D** and the noise matrix **E** using RPCA, i.e.,

(6) $$\begin{array}{c} \begin{array}{cc} \underset{D,E}{\min} & {\left\|D\right\|}_{\text{å}}+\lambda {\left\|E\right\|}_{1}\\ \text{s}.\text{t}. & X=D+E, \end{array} \end{array}$$

where ‖·‖_å_ = ∑ *σ*(·) denotes the nuclear norm, i.e., the sum of its singular values; ‖·‖_1_ = ∑ |·| denotes the *l*_1_-norm, i.e., the sum of its absolute values. Due to **D** represents the clean matrix containing the information of the original data structure and **E** represents the sparse noise matrix, both components are of arbitrary magnitude.

#### 2.2.2. Gene Clustering

As a clustering algorithm based on the concept of message passing, affinity propagation (AP) provides a new method to reveal the inter-relationships between genes. Let *s*(*i*, *k*) be a function that quantifies the similarity between any two genes **x**_*i*_ and **x**_*k*_, let *r*(*i*, *k*) be a function that quantifies how well-suited **x**_*k*_ is to serve as the clustering center for **x**_*i*_, relative to other candidate clustering centers for **x**_*i*_, and let *a*(*i*, *k*) be a function that quantifies how appropriate it would be for **x**_*i*_ to pick **x**_*k*_ as its clustering center, taking into account other points preference for **x**_*k*_ as a clustering center. According to Frey and Dueck in [31], the algorithm performs the following updates iteratively.

First, responsibility updates are sent around:

(7) $$\begin{array}{c} r\left(i,k\right)⟵s\left(i,k\right)-\underset{k^{\prime }\neq k}{\max}\left\{a\left(i,k^{\prime }\right)+s\left(i,k^{\prime }\right)\right\}. \end{array}$$

Then, availability is updated as follows:

(8) ai,k⟵min0,rk,k+∑i′∈i,k max0,ri′,ki≠k,

(9) $$\begin{array}{c} a\left(k,k\right)⟵\underset{i^{\prime }\neq k}{\sum }\;\max\left\{0,r\left(i^{\prime },k\right)\right\}. \end{array}$$

Iterations are performed until either the cluster boundaries remain unchanged over a number of iterations, or some predetermined number of iterations is reached. For the data point *i*, let

(10) $$\begin{array}{c} l=\underset{k}{\arg\;\max}\left\{a\left(i,k\right)+r\left(i,k\right)\right\}, \end{array}$$

if *l* = *i*, then, the data point *i* can be served as a clustering center; otherwise, the data point *l* is seen as a clustering center of *i*. In the implementation of AP clustering, we use negative squared Euclidean distances to measure the similarity, referring to [31] for more details.

Considering that the subtype genes of each cancer may have a specific group structure, the overlapping clustering strategy is adopted, and AP clustering is performed on the data of each cancer to cluster genes. To avoid the influence of noise, the clean data **D** obtained by decomposition is grouped, and the specific process is as follows:

**Step 1 .**

**D** ^**T**^ is divided into *K* sub-matrices depending on different sample labels.

**Step 2 .**

*K* symmetric metric matrices are constructed by using Pearson correlation coefficient.

**Step 3 .**

Based on the above metric matrices, AP clustering is carried out for each kind of data sample to obtain *K* group indicator vectors *v*_1_, *v*_2_, ⋯, *v*_*K*_, the corresponding group sequence of the vector elements specified by the gene.

**Step 4 .**

Expand the dimension of input matrix **D**:

**Step 5 .**

According to *v*_1_, *v*_2_, ⋯, *v*_*K*_, rearrange the columns of **D**, and then get *K* matrices **D**_1_, **D**_2_, ⋯, **D**_*K*_;

**Step 6 .**

The expanded dimension matrix $\bar{D}\in {\mathbb{R}}^{n\times KM}$ is obtained by combining **D**_1_, **D**_2_, ⋯, **D**_*K*_ by row.

**Step 7 .**

The group index vector **v** is constructed according to the specific group sequence in the data matrix after dimensional expansion. Let *V* denotes the maximum value of **v**, i.e., a total of *V* groups are obtained.

#### 2.2.3. Model Construction

Since each gene is repeated *K* times in the expanded dimension matrix $\bar{D}$, to maintain the correspondence between the noise information and the data after dimensional expansion, the noise matrix **E** should be expanded accordingly. As such, rearrange the columns of **E** according to *v*_1_, *v*_2_, ⋯, *v*_*K*_ and then get *K* matrices **E**_1_, **E**_2_, ⋯, **E**_*K*_. The expanded dimension matrix $\bar{E}\in {\mathbb{R}}^{n\times KM}$ is obtained by combining **E**_1_, **E**_2_, ⋯, **E**_*K*_ by row. Obviously, the more noisy the gene is, the less important it is. Without loss of generality, we use the following gene reliability criterion:

(11) $$\begin{array}{c} s_{t}^{\left(l\right)}=\frac{1}{{\left\|{\bar{E}}_{t}^{\left(l\right)}\right\|}_{1}+\varepsilon },t=1,2,\cdots ,K, \end{array}$$

where *s*_*t*_^(*l*)^ represents the importance of the *t*-th gene in the *l*-th group. According to contraction theory, the regression coefficients of more important genes should be given less weight. Therefore, the weight of the *t*-th gene in the *l*-th group can be represented as follows:

(12) $$\begin{array}{c} w_{t}^{\left(l\right)}=\frac{1}{s_{t}^{\left(l\right)}}. \end{array}$$

Further, the weight vector **w** can be obtained.

(13) $$\begin{array}{c} w=\left(w_{1}^{\left(1\right)}\;\cdots \;w_{m_{1}}^{\left(1\right)}\;w_{1}^{\left(2\right)}\;\cdots \;w_{m_{2}}^{\left(2\right)}\;\cdots \;w_{1}^{\left(V\right)}\;\cdots \;w_{m_{V}}^{\left(V\right)}\right). \end{array}$$

For the *K*-classification problem, the regression coefficients corresponding to each discriminant function need to be determined, so a total of *K* × (*KM*) regression coefficients need to be determined. Considering that the coefficients corresponding to the same gene in each discriminant function should have the same weight, the weight vector **w** should be repeated *K* times to obtain *K* identical row vectors, and these *K* identical row vectors can be combined by row to obtain the following *K* dimension weight matrix **W**, i.e.,

(14) $$\begin{array}{c} W={\left(w\;w\;\cdots \;w\right)}^{T}. \end{array}$$

By using noise information to evaluate the importance of each gene, the coefficients in the discriminant function can be adjusted adaptively. In this paper, we introduce the weight matrix **W** into multiclass sparse groups Lasso penalty and establish the following model:

(15) $$\begin{array}{c} P_{\left(\alpha ,\lambda \right)}\left(\beta \right)=\left(1-\alpha \right)\lambda \overset{K}{\underset{k=1}{\sum }}\;\overset{V}{\underset{l=1}{\sum }}\;\sqrt{m_{l}}{\left\|\beta_{l}^{\left(k\right)}\right\|}_{2}+\alpha \lambda \overset{K}{\underset{k=1}{\sum }}\;{\left\|{\left(W\beta^{T}\right)}_{k}\right\|}_{1}, \end{array}$$

where *λ* ≥ 0 and 0 ≤ *α* ≤ 1 are the regularization parameters, *β* = (*β*^(1)^, *β*^(2)^,⋯,*β*^(*K*)^)^*T*^ is the coefficient matrix, and (**W***β*^*T*^)_*k*_ is the *k*-th row of the matrix **W***β*^*T*^.

The multinomial log-likelihood function does not need to presuppose the distribution of the data, and it can directly model the possibility of classification, so the loss function is used to estimate the empirical risk. By introducing adaptive multiclass sparse group Lasso penalty into multinomial log-likelihood function, this paper presents the following robust adaptive multinomial regression with sparse group Lasso penalty (RAMRSGL):

(16) $$\begin{array}{c} \underset{\beta_{0},\beta }{\min}\left\{-\frac{1}{n}\overset{n}{\underset{i=1}{\sum }}\;\left[\overset{K}{\underset{k=1}{\sum }}\;y_{ik}\left(\beta_{0}^{\left(k\right)}+{\bar{x}}_{i}^{T}\beta^{\left(k\right)}\right)-\log\left(\overset{K}{\underset{k=1}{\sum }}\;e^{\beta_{0}^{\left(k\right)}+{\bar{x}}_{i}^{T}\beta^{\left(k\right)}}\right)\right]+P_{\left(\alpha ,\lambda \right)}\left(\beta \right)\right\}. \end{array}$$

where *y*_*ik*_ = *I*(*y*_*i*_ = *k*) is the indicator function, i.e., if the sample belongs to class *k*, *y*_*ik*_ = 1; otherwise, *y*_*ik*_ = 0.

For multiclassification problems, the penalty term $\sum_{k=1}^{K}\;\sum_{l=1}^{V}\;\sqrt{m_{l}}{\left\|\beta_{l}^{\left(k\right)}\right\|}_{2}$ enables RAMRSGL to select important gene groups for all discriminant functions. We also construct data-driven weights using the decomposed noise information, which enables adaptive gene selection within each group. At the same time, by introducing noise information into the model, the robustness of the model is further enhanced.

#### 2.2.4. Solution Algorithm

Solving the group Lasso optimization problem is around for some time, e.g., Similä and Tikka [44] have developed an interesting application to multiresponse linear regression. Due to *l*_1_-norm penalty is not differentiable at the origin, group Lasso algorithms cannot be used to compute a solution to the sparse group Lasso optimization problem. Inspired by Vincent and Hansen [39], we also adopt the algorithm of block coordinate descent, comprising of the outer, middle, and inner coordinate descent loop.

In this work, the proposed RAMRSGL model is used to conduct multiclassification problem and gene selection on the gene expression data of multiple carcinomas. The specific steps are elaborated in Algorithm 1, which is implemented using the R language version of MSGL toolkit (https://github.com/nielsrhansen/msgl) proposed by Vincent and Hansen. The maximum iterations of the proposed RAMRSGL model are set to *i*_max_ = 1000. Moreover, its convergence is proved theoretically, more details in [39].

Although AP clustering does not need to specify the number of clustering in advance, the final number of clustering is affected by the parameter of *p*(*i*), which is the reference degree with the point *i* as clustering center. This means that the higher the value *p*(*i*) is, the greater the possibility of this point becoming the clustering center is. For genes, since each data point has the same possibility of being the clustering center, all *p*(*i*) is set to the same value, which is denoted as *p*.

## 3. Experiments

### 3.1. Dataset

The acute leukemia gene expression dataset is provided by Golub et al. [45], which contains 72 samples consisting of 7129 genes. According to [46], the diagnosis of acute leukemia can be considered as a tri-classification problem, with 38 samples of B-cell acute lymphoblastic leukemia (BALL), 9 samples of T-cell acute lymphoblastic leukemia (TALL), and 25 samples of acute myeloid leukemia (AML). Using the data preprocessing method in [45], 3571 important genes are selected preliminarily. In this paper, preprocessed data is used for the experiment, i.e., a dataset containing 72 samples of 3571 genes. The data is randomly divided into two parts, two-thirds for training and one-third for testing. In order to ensure the class balance of the data, 25 BALL samples, 6 TALL samples, and 17 AML samples are randomly selected as the training set, and the remaining 24 samples are used as the test set.

### 3.2. Clustering Results

**Figure 1:**
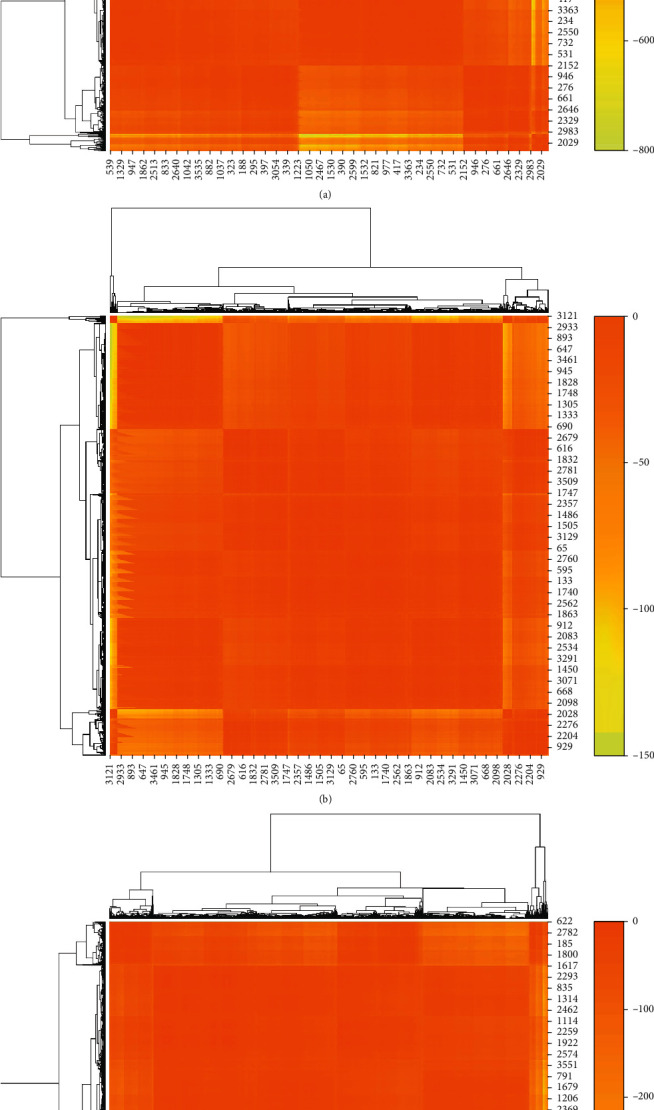
Heatmaps of AP clustering: (a) BALL, (b) TALL, and (c) AML.

AP clustering is performed on BALL, TALL, and AML, respectively, and the heatmaps of AP clustering are shown in Figure 1.  Table 1 elaborates the detailed results of AP clustering. According to the clustering strategy, take the default reference *p* = −26.62758 in BALL class. The 3571 genes are automatically divided into 42 clusters, among which the second cluster has the largest number of genes (252 genes), the first cluster has the smallest number of genes (22 genes), and most of the other clusters have about 100 genes. In TALL class, the default reference *p* = −6.286937 is taken, and the genes are automatically divided into 36 clusters. Among the 36 clusters, the number of genes varies greatly, with the largest cluster containing 347 genes and the smallest cluster containing only 17 genes. In AML class, the default reference *p* = −17.206 is taken, and the genes are divided into 41 clusters, with the largest containing 273 genes and the smallest containing 22 genes. Think of each cluster as a group, and each duplicated gene as a new gene. A total of 10713 genes are obtained by placing the 119 gene groups in a specific order.  Table 1 also reports the fact iterations of the algorithm, from which it can be seen that AP clustering on all datasets can achieve convergence within a finite number of steps.

### 3.3. Performance Comparison

In this paper, the proposed RAMRSGL algorithm is compared with adaptive multinomial regression with sparse group Lasso (AMRSGL), multinomial regression with sparse group Lasso penalty (MRSGL), multinomial regression with group Lasso penalty (MRGL), and multinomial regression with Lasso penalty (*l*_1_-norm MR). RAMRSGL is used to conduct experiments on the clean data obtained by decomposition, and the genes are clustered in advance by AP clustering. The other four methods are all tested on the original data. AMRSGL, MRSGL, and MRGL also used WGCNA for gene clustering, while *l*_1_-norm MR method does not require clustering in advance. The first three of the above methods have two model parameters *α* and *λ* that need to be determined. The last two have only one parameter *λ* to determine.

Table 2 presents the average classification accuracy and average number of selected genes of 10 experiments with different methods on the acute leukemia dataset, with standard deviation in brackets. As can be seen from Table 2, these five methods have achieved high classification accuracy, all reaching more than 94%. The proposed RAMRSGL method has the highest average classification accuracy, 95.8%, which is 0.4%, 0.8%, 0.4%, and 1.2% higher than the other four methods, respectively. AMRSGL and MRGL have achieved a suboptimal classification accuracy of 95.4%, and *l*_1_-norm MR has achieved the lowest classification accuracy of 94.6%. RAMRSGL achieved the smallest standard deviation, indicating that the method is more stable than other methods. It should be noted that the average number of selected genes varies greatly among the five methods. The MRGL method has the most genes selected, with an average number of 571.7. The average number of selected genes was only 21.7 by *l*_1_-norm MR method and 52.2 by RAMRSGL method. To sum up, the proposed RAMRSGL method has the highest classification accuracy and high simplicity, which makes the model easier to be interpreted.

In addition, to further illustrate the robustness of the proposed model and the effectiveness of AP clustering, the experimental results of the three methods on the decomposed clean data are presented in Table 3. Both MRSGL and MRGL use AP clustering to cluster genes in advance, while *l*_1_-norm MR does not cluster. The parameter selection method is consistent with the above. As can be seen from Table 3, the classification accuracy of these three methods on clean data is higher than before, and the variance is relatively small. It is proved that the use of robust principal component analysis can improve the model performance. Otherwise, the deviation of the prediction accuracy in Table 3 is higher than the proposed method in Table 2, which proves that the proposed method using AP clustering is more robust. In terms of gene selection, the experimental results of MRSGL and MRGL methods are significantly different from those of the previous ones, with the average number of selected genes being significantly reduced. The ability to achieve high accuracy with fewer genes is very attractive. After AP clustering is used for gene clustering in advance, fewer genes are selected by these methods, which may benefit from the fact that AP clustering can well reveal the group structure between genes, so as to achieve more accurate gene selection.

### 3.4. Gene Selection

In each experiment, as can be seen in Algorithm 1, we extract the nonzero coefficients *β*^⋆^ of the optimal model and determine the corresponding genes and groups. By selecting genes that appear 9 or more times in 10 experiments as key genes, RAMRSGL has identified 9 key genes on the leukemia dataset. Seven gene replicates are selected for 10 times, and two gene replicates are selected for 9 times.  Table 4 lists seven genes and their corresponding group sequences that are present in each experiment. In addition, through the search of these genes in the NCBI database, the functional annotations of these 7 genes are also given in Table 4.

**Figure 2:**
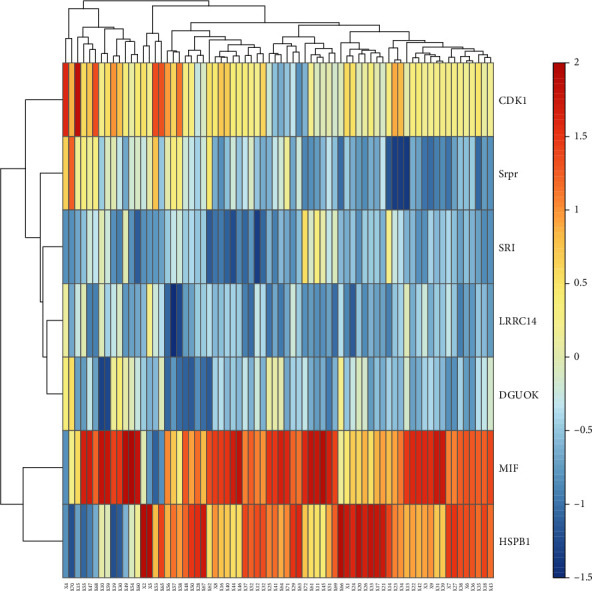
Clustering heat map of seven selected genes.

**Figure 3:**
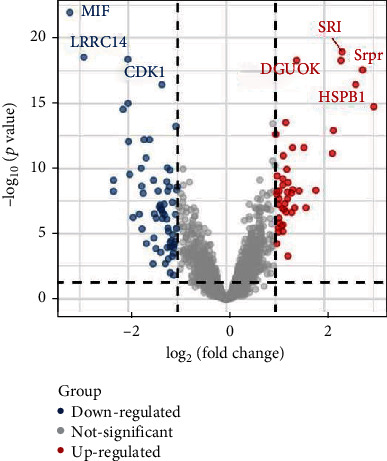
Volcano plot for differential expression of selected genes.

**Figure 4:**
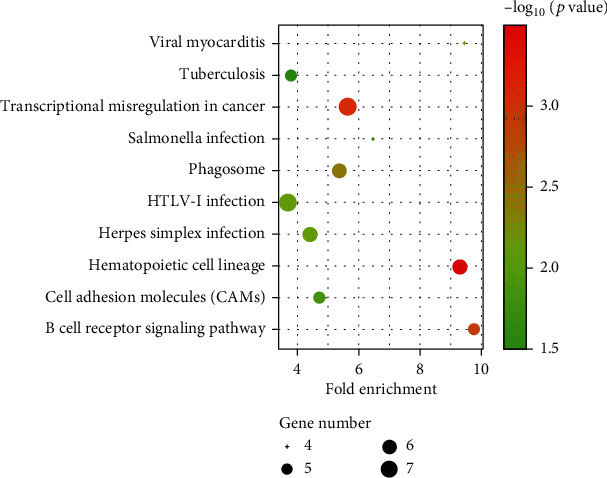
Bubble diagram of selected genes.

Figure 2 shows the heat map of the selected seven genes in different samples. It can be seen that genes HSPB1 and MIF have similar expressions in different samples and can be grouped into one group, while Srpr, SRI, DGUOK, and LRRC14 can be grouped into another group. It is concluded that these genes have similar functions or are jointly involved in some gene pathways.  Figure 3 illustrates the volcano plot for differential expression of 3571 genes where the differentially expressed genes are selected by the threshold *p* < 0.05(log_2_FC < 1 and log_2_FC>−1). As can be seen from Figure 3, among the 7 screened genes, the expressions of SRI, Srpr, HSPB1, and DGUOK are significantly upregulated, while the expressions of MIF, LRRC14, and CDK1 are significantly downregulated. The bubble diagram of genes selected in an experiment is shown in Figure 4. It can be seen from Figure 4 that the cell pathways involved in these genes mainly include viral myocarditis, tuberculosis, transcription disorders in cancer, hematopoietic cell lineage, and B cell signaling pathway. In addition, more of these genes are involved in cancer transcription disorders, and more are involved in viral infection.

Combined with gene function and literature, the relationship between five key genes and cancer is expounded.

#### 3.4.1. DGUOK

The protein encoded by this gene is responsible for the phosphorylation of purine deoxyribonucleosides in the mitochondrial matrix. This protein phosphorylates several purine deoxyribonucleoside analogs used in the treatment of lymphoproliferative disorders, and this phosphorylation is critical for the effectiveness of the analogs. Wu et al. [47] have found that DGUOK-AS1 is upregulated in cervical squamous cell carcinoma and intracervical adenocarcinoma (CESC) tissues. Their research has also shown that DGUOK-AS1 is highly expressed in liver cancer cell lines and can promote the proliferation of cervical cancer cells by releasing EMSY as the ceRNA of miR-653-5p.

#### 3.4.2. MIF

This gene encodes a lymphokine involved in cell-mediated immunity, immune regulation, and inflammation. By inhibiting the anti-inflammatory effects of glucocorticoids, it regulates the function of macrophages in host defense. Osipyan et al. [48] have found that MIF can trigger the mitogen-activated protein kinase and phosphoinosinic acid 3-kinase signaling pathways by binding to CD74 and other receptors. The change in the expression value of MIF and changes in the active state of connection pathways are related to inflammatory diseases and cancer.

#### 3.4.3. CDK1

The protein encoded by this gene is a member of the Ser/Thr protein kinase family. This protein is the catalytic subunit of the highly conserved protein kinase complex M-phase promoting factor (MPF), which plays an important role in the transition of the G1/S and G2/M phases of the eukaryotic cell cycle. The mitotic cyclin binds to the protein stably and functions as a regulatory subunit. The phosphorylation and dephosphorylation of this protein also play an important regulatory role in cell cycle control. Huang et al. [49] have studied the mechanism of CDK1 in lung cancer and found that CDK1 is regulated by NF-KB through a hypothetical KB site in its proximal promoter.

#### 3.4.4. Srpr

This gene encodes a subunit of the endoplasmic reticulum signal recognition particle receptor, and together with the signal recognition particle, it participates in the targeting and translocation of secreted proteins and cell membrane proteins marked by signal sequences. Alternative splicing leads to multiple transcriptional variations. Kim et al. [50] have found that Srpr is highly expressed in epidermal keratinocytes and regulates the proliferation of keratinocytes by affecting cell cycle progression.

#### 3.4.5. HSPB1

This gene encodes a protein of the small heat shock protein (HSP20) family. This protein plays an important role in the differentiation of many cell types. The expression of this gene is associated with the adverse clinical outcomes of a variety of human cancers. The encoded protein can promote the proliferation and metastasis of cancer cells, while protecting cancer cells from apoptosis. Rajesh et al. [51] have established Fli-1 (Fli-1), a member of the Ets family, which plays a transcriptional regulatory role on the HSPB1 gene. Fli-1 binds to the nucleotide residues GGAA at binding sites 3, 6, and 7 in the 5-kb region upstream of HSPB1. Fli-1 is related to oncogenic transformation and upregulation in radio/TMZR GBM.

## 4. Conclusions

In this paper, a robust adaptive multinomial regression model with sparse group Lasso penalty is proposed and its solution algorithm is developed, based on robust principal component analysis and AP clustering with overlapping strategy. The proposed method is applied to the diagnosis of triple-cancer leukemia, and the accuracy of diagnosis is up to 95.8%, which is better than other state-of-the-art methods. In addition, seven key genes are screened out, and the relationship between five key genes and cancer is expounded in combination with gene function and relative literature. In the future, the nonlinear problem will be studied.

## Figures and Tables

**Algorithm 1:**
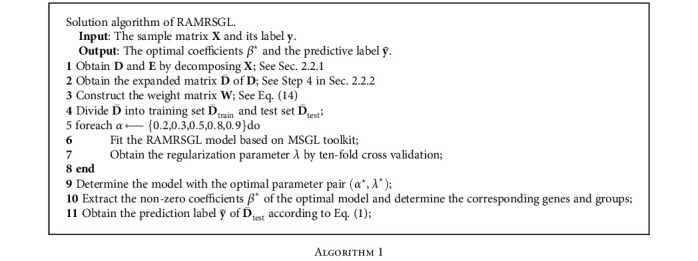


**Table 1:** Results of AP clustering on BALL, TALL, and AML.

Data	BALL	TALL	AML
Number of iterations	175	151	199
*p* value	-26.62758	-6.286937	-17.206
Sum of similarities	-3108.519	-449.0737	-2029.779
Net similarity	-4226.878	-675.4035	-2735.225
Number of clusters	42	36	41

**Table 2:** Performance of five methods on the leukemia dataset.

Methods	Average prediction accuracy	Average number of selected genes
RAMRSGL	0.958 (0.026)	52.2 (26.01)
AMRSGL	0.954 (0.043)	375.1 (119.66)
MRSGL	0.950 (0.036)	239.5 (122.20)
MRGL	0.954 (0.051)	571.7 (221.08)
*l*_1_-norm MR	0.946 (0.049)	21.7 (5.97)

**Table 3:** Classification accuracy and number of selected genes on the decomposed clean data.

Methods	Average prediction accuracy	Average number of selected genes
MRSGL	0.954 (0.039)	126.2 (34.25)
MRGL	0.958 (0.032)	160.2 (53.72)
*l*_1_-norm MR	0.958 (0.037)	18.9 (3.24)

**Table 4:** Seven key genes selected by RAMRSGL on leukemia data.

Gene	Number group	Gene title	Annotation of gene function
CDK1	2	Cyclin-dependent kinase 1	Phosphorylation and dephosphorylation of CDK1-encoded proteins play an important role in cell cycle regulation.
LRRC14	24, 65	Leucine-rich repeat containing 14	LRRC14 negatively regulates NF-kappa B transcription factor activity and toll-like receptor signaling pathway.
MIF	51	Macrophage migration inhibitory factor	MIF encodes a lymphokine involved in cell-mediated immunity, immunomodulation, and inflammation.
Srpr	84	Signal recognition particle receptor	Srpr plays a role in signal recognition of particle binding.
DGUOK	46	Deoxyguanosine kinase	The protein encoded by DGUOK is responsible for phosphorylation of purine DNA in the mitochondrial matrix.
SRI	88	Sorcin	SRI encodes a calcium-binding protein that regulates intracellular calcium homeostasis.
HSPB1	105	Heat shock protein family B member 1	HSPB1 encodes proteins that play important roles in the differentiation of a variety of cell types.

## Data Availability

The acute leukemia gene expression dataset is provided by Golub et al., which can be download at https://www.kaggle.com/crawford/gene-expression.
